# Edward Jenner's Discovery of Vaccination: Impact and Legacy

**DOI:** 10.7759/cureus.68993

**Published:** 2024-09-09

**Authors:** Niranjan Raja, Aarthi Ashwinth Jothy

**Affiliations:** 1 Department of Nephrology, Mahatma Gandhi Medical College and Research Institute, Puducherry, IND

**Keywords:** edward jenner, historical vignette, public health, smallpox, vaccination

## Abstract

Edward Jenner's pioneering work in the late 18th century, particularly his development of the smallpox vaccine, marked a transformative moment in medical history. This review meticulously examines Jenner's groundbreaking discovery and its profound impact on modern immunology and public health. By analyzing a comprehensive range of peer-reviewed studies and historical accounts, this review provides an in-depth exploration of Jenner's experimental methodology, the scientific and ethical controversies that surrounded his work, and the subsequent adoption of vaccination practices. Jenner’s rigorous approach to scientific inquiry, the broader implications of his work on public health strategies, and its enduring relevance in addressing contemporary health challenges are also highlighted. This comprehensive analysis underscores Jenner's legacy as a cornerstone of modern medicine and public health.

## Introduction and background

Introduction

The discovery of vaccination by Edward Jenner in the late 18th century represents a watershed moment in the history of medicine and public health. Prior to Jenner’s groundbreaking work, smallpox was a pervasive and devastating disease that caused significant morbidity and mortality worldwide [1]. The development of the smallpox vaccine not only transformed the approach to combating infectious diseases but also laid the groundwork for the modern field of immunology.

Background

Smallpox, caused by the variola virus, was a major global health threat for centuries. The disease was characterized by high fever, malaise, and a distinctive rash that often led to severe complications and death [2]. Historical records suggest that smallpox was responsible for significant epidemics throughout human history, with profound social and economic impacts. The prevalence of smallpox created a pressing need for effective preventive measures, which catalyzed the search for a viable solution [1].

Edward Jenner was born on 17 May 1749, at the vicarage, Berkeley, in Gloucestershire [3]. Jenner’s work in the context of vaccination was revolutionary. Prior to him, various methods for preventing smallpox had been attempted, including variolation. Variolation involved the deliberate inoculation of smallpox material into healthy individuals to induce a mild form of the disease and provide immunity. Although variolation provided some level of protection, it was associated with significant risks, including severe disease and death [4]. Jenner’s approach offered a safer and more effective alternative, fundamentally changing the trajectory of infectious disease prevention.

## Review

Jenner’s contributions and innovations

Edward Jenner, a country physician with keen observational skills, noticed that milkmaids who had contracted cowpox, a less severe disease caused by the cowpox virus, seemed to be immune to smallpox [5]. This observation led Jenner to hypothesize that cowpox could be used to confer immunity against smallpox. In 1796, Jenner conducted a landmark experiment in which he inoculated a young boy, James Phipps, with material taken from a cowpox lesion. Subsequently, when Phipps was exposed to smallpox, he did not develop the disease. This experiment demonstrated that cowpox could indeed provide protection against smallpox and established the basis for vaccination [6].

Jenner’s innovation was not merely in his experimental technique but also in his rigorous approach to scientific inquiry. He meticulously documented his findings and published them in his seminal work, "An Inquiry into the Causes and Effects of the Variolae Vaccinae," in 1798. This publication provided a comprehensive account of his observations, experimental procedures, and the evidence supporting the efficacy of vaccination [5]. Jenner’s detailed documentation and methodical validation of his results were crucial in establishing the scientific basis for vaccination.

Impact and adoption

The immediate impact of Jenner’s discovery was substantial, but it also faced significant challenges. Initially, Jenner’s methods encountered skepticism from the medical community and the public. Critics questioned the safety and efficacy of vaccination, and there were ethical concerns regarding the use of cowpox material [7]. Despite these obstacles, Jenner’s persistence and the growing body of evidence supporting vaccination led to its gradual acceptance. By the early 19th century, vaccination began to gain broader acceptance, leading to the implementation of vaccination programs and a dramatic reduction in the incidence of smallpox [8].

The successful introduction of vaccination had far-reaching implications beyond smallpox. Jenner’s principles of immunization paved the way for the development of vaccines against other infectious diseases, such as measles, polio, and influenza [9]. The methods and insights gained from Jenner’s work have continued to influence vaccine development and public health strategies [10].

Legacy and modern relevance

Jenner’s discovery laid the foundation for the modern field of immunology and the global vaccination efforts that have since become integral to public health [11]. The eradication of smallpox, achieved through extensive vaccination campaigns, stands as one of the most significant achievements in medical history and a testament to the effectiveness of vaccination as a public health tool [12].

In contemporary times, the principles established by Jenner continue to guide vaccine development and public health initiatives. The lessons learned from Jenner’s work remain relevant as new vaccines are developed and as public health professionals address emerging challenges, such as vaccine hesitancy and the need for vaccines against newly identified pathogens [13]. Jenner’s legacy underscores the importance of scientific innovation, rigorous research, and the continued pursuit of effective strategies to combat infectious diseases.

**Figure 1:**
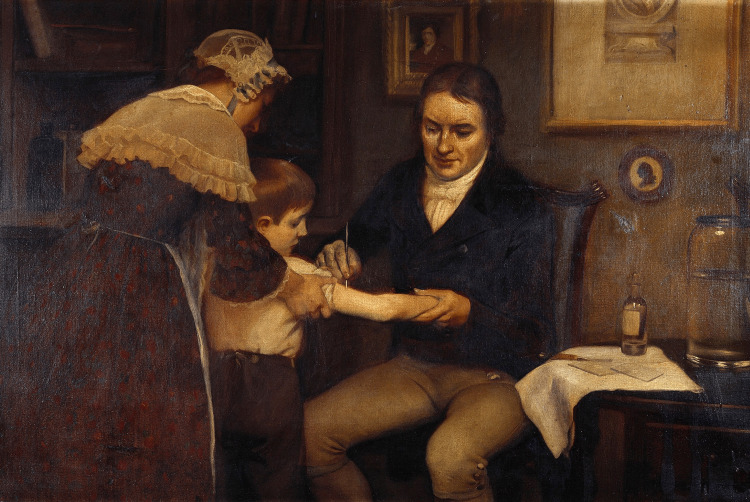
Jenner performing his first vaccination, 1796 Image credits: [14] Image used under the Creative Commons Attribution 4.0 International license.

This review aims to provide a comprehensive examination of Edward Jenner’s contributions, exploring the historical context, key studies, impact on public health, and ongoing relevance of his work. Figure 1 is a painting showing an artist's impression of Dr. Jenner performing his first vaccination.

Methods

A comprehensive literature search was conducted on PubMed using keywords such as "Edward Jenner," "smallpox vaccination," "history of immunization," and "vaccination impact." The search was limited to articles published in peer-reviewed journals. Inclusion criteria were based on the relevance to Jenner's work, methodological rigor, and contribution to understanding the discovery and its implications. More than 20 articles were included in this review, providing a broad perspective on Jenner’s work and its legacy.

Historical context

Early Observations and Jenner’s Hypothesis

Edward Jenner’s observations and hypotheses were rooted in his experiences as a country physician and his interest in the natural world. His recognition of the relationship between cowpox and smallpox immunity was informed by the folk knowledge of the time and his own clinical observations [1]. Jenner’s hypothesis was that cowpox, a less virulent disease, could induce immunity to smallpox, a much more severe disease. Jenner’s experiment in 1796, which involved inoculating James Phipps with cowpox and subsequently exposing him to smallpox, was designed to test this hypothesis. The success of this experiment provided the first evidence that vaccination with cowpox could indeed protect against smallpox [6]. Jenner’s work was groundbreaking not only because of the results but also because of the rigorous scientific method he employed.

Early Skepticism and Adoption

Jenner’s discovery faced considerable skepticism from contemporary medical and scientific communities. Critics questioned the validity of his results, the safety of the procedure, and the ethical implications of using animal-derived material for human vaccination [15]. Despite these challenges, Jenner’s meticulous documentation and the growing body of evidence supporting the efficacy of vaccination led to its gradual acceptance. The early 19th century saw the beginning of widespread vaccination programs. Governments and public health organizations began to recognize the potential of vaccination to control infectious diseases. The establishment of vaccination protocols and the promotion of vaccination campaigns contributed to a significant decline in smallpox incidence and mortality leading to the elimination of the disease in 1980 by WHO [16].

Key studies and findings

Jenner’s Original Publications

Jenner’s seminal publication, "An Inquiry into the Causes and Effects of the Variolae Vaccinae," was a comprehensive account of his experiments and observations. This work provided detailed descriptions of Jenner’s methodology, including the process of inoculation with cowpox and the subsequent exposure to smallpox [5]. The publication was critical in establishing the scientific basis for vaccination and garnered widespread attention. The methodological rigor demonstrated in Jenner’s publication was instrumental in addressing the skepticism surrounding vaccination. Jenner’s careful documentation of his experiments and the presentation of evidence supporting the efficacy of vaccination played a key role in validating his work [17].

Impact on Public Health

The introduction of vaccination had a profound impact on public health. The decline in smallpox incidence and mortality following the widespread adoption of vaccination is well-known. During the 20th century, significant achievements have been made in the control of many vaccine-preventable diseases. Nine vaccine-preventable diseases and their complications which included smallpox, along with the eight diseases had been recommended for universal vaccination in children as of 1990 out of them smallpox has been eradicated and poliomyelitis caused has been eliminated [18]. The success of smallpox vaccination led to the development of vaccines for other infectious diseases. The principles of vaccination established by Jenner have been applied to the development of vaccines for diseases such as measles, polio, and influenza. The continued success of vaccination programs is a testament to the enduring influence of Jenner’s work [19].

Ethical and Scientific Considerations

Jenner’s work was subject to ethical and scientific scrutiny. Early critiques focused on the safety and ethical implications of using cowpox for vaccination. Concerns were raised about the potential long-term effects of vaccination and the morality of using animal-derived material [20]. Jenner’s work eventually overcame these objections through rigorous scientific validation and the demonstration of the benefits of vaccination. The resolution of these ethical concerns and the validation of Jenner’s findings were crucial in the widespread adoption of vaccination. The ethical and scientific considerations surrounding Jenner’s work continue to inform discussions on vaccine safety and efficacy today.

Legacy and modern implications

Advances in Vaccination

Jenner’s discovery set a precedent for the development of vaccines against various infectious diseases. Modern vaccine development builds on Jenner’s principles, utilizing advanced techniques such as recombinant DNA technology, adjuvants, and novel delivery systems to enhance vaccine efficacy and safety [21]. The development of vaccines for emerging infectious diseases, such as Ebola and COVID-19, reflects the continued relevance of Jenner’s principles [9]. The success of modern vaccines demonstrates the ongoing impact of Jenner’s work on public health. Advances in vaccine technology and the application of Jenner’s principles continue to play a critical role in controlling and preventing infectious diseases [22].

Impact on Immunology

Jenner’s work revolutionized the field of immunology. The concept of vaccination introduced by Jenner led to the establishment of key principles in immunology, including the concept of acquired immunity and the development of preventive medicine strategies. Jenner’s discovery provided the foundation for understanding how the immune system can be stimulated to provide protection against infectious diseases. The influence of Jenner’s work on modern immunology is profound. It has shaped both basic research and clinical practice, guiding the development of new vaccines and immunization strategies. The principles established by Jenner continue to inform research into immune responses and vaccine development [6].

Public Health Strategies

The success of smallpox vaccination demonstrated the potential of vaccination programs to control and eliminate infectious diseases. The global smallpox eradication campaign, led by the WHO, is a testament to the effectiveness of vaccination as a public health tool [23]. The principles of mass vaccination, surveillance, and containment developed during the smallpox eradication effort continue to inform public health strategies for other diseases. Public health initiatives, such as vaccination campaigns for influenza, human papillomavirus (HPV), and COVID-19, build on the lessons learned from the smallpox eradication effort. These initiatives aim to achieve high vaccination coverage and reduce the incidence of infectious diseases.

Discussion

Lessons from Jenner’s Work

Jenner’s work provides several important lessons for modern public health. Firstly, it underscores the importance of rigorous scientific evaluation and validation of new medical interventions. Jenner’s meticulous documentation and experimentation were crucial in demonstrating the efficacy of vaccination. His approach to scientific inquiry and evidence-based practice continues to be relevant in contemporary research and public health. Secondly, the challenges faced by Jenner highlight the need for clear communication and public engagement in the face of new health interventions. Addressing concerns and educating the public about the benefits and safety of vaccination are essential for achieving high vaccination coverage and preventing disease outbreaks [24].

Ongoing Challenges and Future Directions

While Jenner’s work laid the foundation for modern vaccination practices, challenges remain in the field of immunology. Vaccine hesitancy, emerging infectious diseases, and the need for new vaccines for diseases with complex epidemiological patterns require ongoing research and innovation. Future research should focus on improving vaccine technology, understanding immune responses, and addressing public concerns about vaccination [25]. The development of new vaccines and vaccination strategies will be essential for addressing future public health challenges. Collaboration between researchers, policymakers, and public health officials will be crucial for advancing vaccination efforts and ensuring the continued success of immunization programs [26].

## Conclusions

Edward Jenner’s discovery of vaccination represents a transformative moment in medical history. His work laid the groundwork for the development of modern immunology and public health strategies. This review highlights the significant impact of Jenner’s work on the prevention of infectious diseases and its enduring relevance in contemporary medicine. As we continue to face new health challenges, Jenner’s legacy remains a testament to the power of scientific innovation and its potential to improve global health.
